# Interpreting combined BALF-galactomannan and next-generation sequencing in pulmonary aspergillosis and mucormycosis: a two-center clinical decision framework

**DOI:** 10.3389/fmicb.2026.1873197

**Published:** 2026-07-22

**Authors:** Lan Yang, Xiaoqi Zhang, Nan Su, Lijuan Li

**Affiliations:** 1 Department of Respiratory and Critical Care Medicine, Second Affiliated Hospital of Harbin Medical University, Harbin, China; 2 Department of Pulmonary and Critical Care Medicine, Second People’s Hospital of Weifang, Weifang, China; 3 Department of Pulmonary and Critical Care Medicine, National Centre for Clinical Research on Respiratory Diseases, China-Japan Friendship Hospital, Beijing, China

**Keywords:** bronchoalveolar lavage fluid galactomannan, diagnostic concordance, immunocompromised host, next-generation sequencing, pulmonary aspergillosis, severe pneumonia

## Abstract

**Objectives:**

To identify which patients with pulmonary aspergillosis or mucormycosis (PAM) derive the greatest incremental diagnostic benefit from next-generation sequencing (NGS) beyond conventional microbiology, and to derive a GM × NGS interpretive framework that identifies the patient subgroup in which NGS adds the most information, based on routinely available bedside variables. Secondary objectives were to characterize the pathogen spectrum detected by NGS, quantify pairwise concordance with BALF galactomannan and culture, and assess the relationship between quantitative Aspergillus reads and BALF-GM intensity.

**Methods:**

Two-center retrospective case-cohort study of 595 adults with NGS-tested pulmonary aspergillosis or mucormycosis (PAM; proven, probable, or possible per EORTC/MSGERC and ECMM-MSGERC criteria). Primary method-comparison analysis was restricted to the strict subset with NGS, BALF-galactomannan (GM), and culture all available (*n* = 434). Total Aspergillus reads were defined as the per-case sum of species-level Aspergillus reads parsed from free-text NGS reports, with Mucorales reads excluded. Multivariable logistic regression identified predictors of GM-negative status among NGS-positive cases (*n* = 353; complete-case *n* = 342). The prospective component was registered at the Chinese Clinical Trial Registry (ChiCTR2500099900).

**Results:**

In the strict 434-case subset, NGS fungal-signal positivity was 81.3%, BALF-GM (≥0.8) 58.1%, and culture 43.1%; pairwise overlap was substantial but incomplete. The GM-negative/NGS-positive subgroup (*n* = 150) showed a milder phenotype than GM-positive/NGS-positive cases (severe pneumonia 28.7% (43/150) vs. 52.7% (107/203); mortality 6.7% (10/150) vs. 19.7% (40/203); both *p* < 0.001). Severe pneumonia was independently inversely associated with GM-negative status (adjusted OR 0.44, 95% CI 0.26–0.75, *p* = 0.002), while immunocompromise was independently positively associated (adjusted OR 2.01, 95% CI 1.17–3.47, *p* = 0.012). NGS Aspergillus positivity was essentially flat across BALF-GM intensity bands (79–82%); read counts correlated only weakly with BALF-GM titer [Spearman ρ = 0.185 (mNGS-only, *n* = 246)].

**Conclusion:**

When BALF-GM and NGS are co-ordered on the same lavage in routine PAM workup, the four GM × NGS quadrants define distinct clinical phenotypes with concrete management implications: NGS positivity in GM-negative cases (34.6%) carries the highest incremental yield and a milder phenotype; conversely, a negative NGS in GM-positive cases must not be used to rule out severe PAM. Across quadrants, NGS adds species- and co-pathogen-level information that changes therapy.

## Background

Pulmonary aspergillosis and mucormycosis (PAM) are microbiologically challenging infections, including influenza- and COVID-19-associated forms in critically ill hosts (Cornely et al., 2019; Donnelly et al., 2020; Koehler et al., 2021; Patterson et al., 2016; Verweij et al., 2020), in which conventional methods—culture, galactomannan (GM), serology, and histopathology—each have well-characterized limitations (Cornely et al., 2019; Donnelly et al., 2020; Lass-Flörl et al., 2021; Patterson et al., 2016; Skiada et al., 2018). Culture is insensitive and slow; GM performance varies by host and specimen (Mercier et al., 2021); histopathology is highly specific but rarely available; alternative immunoassays and Aspergillus PCR have been evaluated to address these gaps (Eigl et al., 2015; Heldt et al., 2018; Imbert et al., 2018). Respiratory samples in PAM frequently contain multiple microbial signals, complicating interpretation in routine practice.

Metagenomic and targeted next-generation sequencing (mNGS/tNGS) detect fungi, bacteria, and viruses simultaneously and are increasingly applied in complex pulmonary infections (Chen et al., 2021; Chen et al., 2022; Diao et al., 2021; Guo et al., 2024; Sun et al., 2021) and other infectious syndromes such as Pneumocystis jirovecii pneumonia (Jiang et al., 2021); molecular identification has also strengthened diagnosis of mucormycosis (Muthu et al., 2025). However, much of the existing literature has framed NGS as a diagnostic-performance tool, comparing positivity rates between selected groups or claiming superiority over conventional microbiology (Chen et al., 2022; Guo et al., 2024; Xu et al., 2023). Such framing is hard to interpret when NGS testing is ordered selectively, reference standards are heterogeneous, and many detected organisms may represent co-infection, colonization, contamination, or clinically uncertain signals.

We therefore approached NGS from a different angle: rather than asking whether NGS is globally superior, we asked what microbiological structure NGS reveals within an established PAM cohort, and how those findings align with conventional microbiology. In a two-center case cohort of adults with PAM, we (i) described the pathogen spectrum detected by mNGS/tNGS, (ii) quantified pairwise concordance with culture, BALF-GM, and pathology in patients with all three methods available, and (iii) characterized which patients gain the most incremental information from NGS, with attention to GM-negative/NGS-positive cases as a specific clinical scenario.

## Materials and methods

### Study design and population

This was a two-center retrospective case-cohort study of adults with PAM who underwent NGS testing (proven, probable, or possible per the 2020 EORTC/MSGERC criteria for invasive aspergillosis (Donnelly et al., 2020) and the 2019 ECMM-MSGERC consensus criteria for mucormycosis (Cornely et al., 2019)), managed at China-Japan Friendship Hospital and Second People’s Hospital of Weifang between February 2019 and May 2025. Reporting follows the STROBE recommendations for observational studies (von Elm et al., 2007). The prospective component of this study was registered at the Chinese Clinical Trial Registry (ChiCTR2500099900; registered 31 March 2025) prior to patient enrolment.

### Diagnostic criteria

Diagnoses were assigned per the 2020 EORTC/MSGERC consensus criteria (Donnelly et al., 2020). Chronic pulmonary aspergillosis was diagnosed on the basis of persistent clinical symptoms, characteristic radiological findings (e.g., lung cavities, aspergillomas), and serological evidence of Aspergillus infection. Aspergillus-associated airway diseases included non-invasive airway disorders such as allergic bronchopulmonary aspergillosis (ABPA) and other chronic airway diseases with Aspergillus involvement. Mucormycosis cases were classified per the 2019 ECMM-MSGERC criteria (Cornely et al., 2019).

### NGS ascertainment and parsing

**FIGURE 1:**
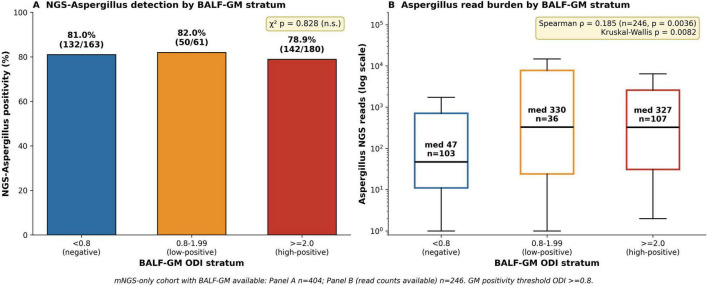
BALF-GM ODI stratified analysis of NGS Aspergillus signal (mNGS only). **(A)** Grouping now uses ODI bands <0.8 / 0.8-1.99 / > = 2.0 with positivity 81.0% / 82.0% / 78.9% (*N* = 404; chi-square *p* = 0.828). **(B)** Read-count medians 47 / 330 / 327 (reads-available *n* = 246; *n* = 103 / 36 / 107; Spearman rho = 0.185, *p* = 0.0036; Kruskal-Wallis *p* = 0.0082). Read-count analyses are restricted to the single platform (mNGS) with comparable read output.

NGS (mNGS or tNGS) was performed when ordered by treating clinicians. tNGS was performed by KingMed Diagnostics (Guangzhou, China); mNGS was performed primarily by Dinfectome Inc. (Nanjing, China); a small number of cases (< 10) used additional mNGS providers (Weiyuan Biotechnology and Weiyan Biotechnology). All mNGS providers used standardized nucleic-acid extraction, library preparation, and pathogen-database alignment workflows; for analyses that depend on intra-platform read-count comparability (Figure 1 and Supplementary Tables S1, S2), only Dinfectome cases were retained. From each free-text report we extracted (i) qualitative organism flags (Aspergillus, Mucorales, bacterial, viral, Candida/yeast, Pneumocystis) and (ii) species-level read counts. All qualitative organism flags were independently verified by two-author manual review of the full 595-case NGS report set, with 100% concordance with the automated parser. Aspergillus read counts were extracted at the species level from NGS reports. Quantitative analyses were stratified by NGS technology (mNGS vs. tNGS) and never pooled across technologies, given fundamental differences in genomic coverage and library normalization between targeted (tNGS) and metagenomic (mNGS) workflows. Within the strict 434-case subset, reads were available for 246 of 353 NGS-positive cases (69.7%). Reads-missing cases were concentrated in earlier-period mNGS reports in which species-level read counts were not enumerated in the free-text output, irrespective of parsing approach. Parsing details are provided in Supplementary methods.

#### BALF-galactomannan testing

BALF-galactomannan was measured locally by Platelia Aspergillus EIA (Bio-Rad) at both centers. Per institutional convention and consistent with published Chinese tertiary practice, BALF-GM positivity was defined as an optical density index (ODI) ≥ 0.8, balancing the EORTC/MSGERC reference threshold of ≥ 1.0 against published evidence supporting lower BALF cut-offs in non-neutropenic and mixed-risk populations. Sensitivity analyses at alternative BALF-GM ODI thresholds (≥ 0.5 and ≥ 1.0) are presented in the Results. Serum galactomannan was also measured by the same Platelia EIA (positivity threshold ODI ≥ 0.5, per the EORTC/MSGERC reference standard for serum). Because BALF-GM provides a higher-yield local signal for pulmonary disease and because the present analysis focuses on same-specimen BAL-based diagnostics (NGS, BALF-GM, and culture all performed on a single lavage), serum GM was not used in the primary classification.

### Conventional microbiology

Conventional microbiology comprised fungal culture from respiratory specimens, BALF galactomannan (Platelia Aspergillus EIA, Bio-Rad), serum galactomannan, Aspergillus IgG serology, and histopathology when biopsy was performed. Respiratory viruses (SARS-CoV-2, cytomegalovirus, RSV, influenza A/B, parainfluenza, human rhinovirus, adenovirus) were detected by RT-PCR.

### Primary analytic subset

To minimize selection bias inherent to NGS test ordering, the primary method-comparison analyses were restricted to cases with NGS, BALF-GM, and culture all available (strict subset, *n* = 434).

### Statistical analysis

Categorical variables are expressed as frequency (percentage) and were compared by Fisher exact or chi-square tests; continuous variables are expressed as median (interquartile range) and were compared by Mann-Whitney U or Kruskal-Wallis tests. Multivariable logistic regression for GM-negative status among NGS-Aspergillus-positive cases (*n* = 353; complete-case *n* = 342) included 10 pre-specified covariates: age > 65 years, bacterial co-detection, viral co-detection, severe pneumonia, chronic kidney disease, immunocompromised status, immunosuppressant use, inhaled corticosteroids, cavity, and reticular/fibrotic bands. Leukemia was evaluated in a sensitivity analysis. Mucormycosis was excluded from the multivariable model owing to near-perfect separation. Spearman and log-log Pearson correlations quantified the read-GM relationship. Two-sided *p* < 0.05 was considered statistically significant. Analyses were performed in Python 3.12 (pandas 2.3, numpy 2.4, scipy 1.17, statsmodels 0.14).

## Results

### Pathogen spectrum

**FIGURE 2:**
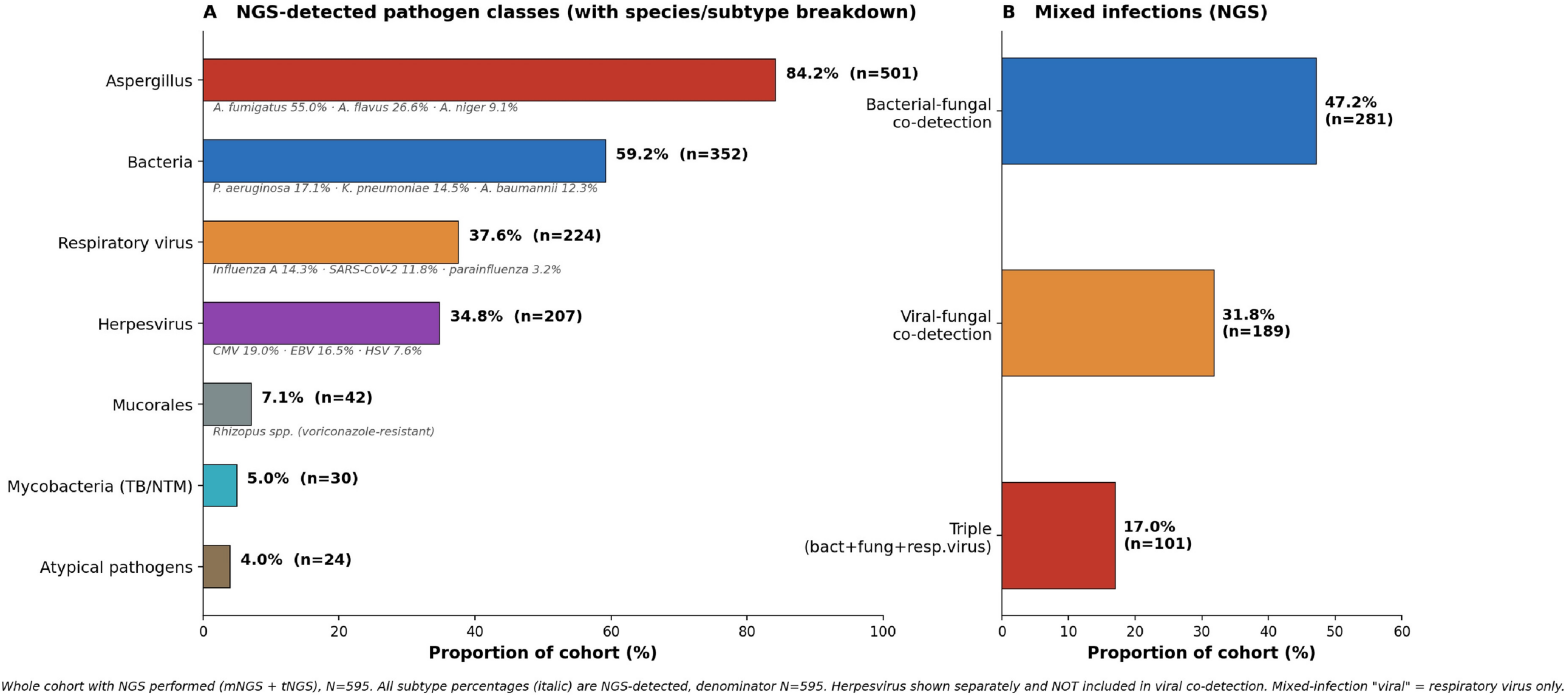
NGS-detected pathogen landscape in the PAM cohort (*N* = 595). Respiratory virus and herpesvirus are now shown as two separate bars (respiratory virus 224, 37.6%; herpesvirus 207, 34.8%) rather than a single combined viral bar. Aspergillus 501 (84.2%), Mucorales 42 (7.1%), Bacteria 352 (59.2%), Mycobacteria 30 (5.0%), Atypical 24 (4.0%); mixed bacterial+fungal 281 (47.2%), respiratory-viral+fungal 189 (31.8%), triple 101 (17.0%).

In the NGS-tested subset (*n* = 595), the dominant Aspergillus species captured by NGS or culture were A. fumigatus 55.0% (327/595), A. flavus 26.6% (158/595), A. niger 9.1% (54/595), A. oryzae 4.0% (24/595), A. nidulans 3.0% (18/595), and A. terreus 2.4% (14/595). Mucorales (predominantly Rhizopus) were detected in 7.1% (42/595) of cases. NGS-detected co-signals were common: bacterial 59.2% (352/595)—with Pseudomonas aeruginosa 17.1% (102/595), Klebsiella pneumoniae 14.5% (86/595), and Acinetobacter baumannii 12.3% (73/595) as the leading bacterial co-pathogens; respiratory viral co-signal 37.6% (224/595) and herpesvirus co-signal 34.8% (207/595), with cytomegalovirus the most frequent (CMV 19.0%, 113/595), followed by Epstein-Barr virus (EBV 16.5%, 98/595) and herpes simplex virus (HSV 7.6%, 45/595). Mixed-category (≥ 2) signals occurred in 60.3% of cases, underscoring the polymicrobial structure routinely revealed by NGS in this population (Figure 2).

### Cohort and NGS coverage

The study cohort comprised 595 adult PAM cases with NGS testing drawn from a source population of 1,042 adults under evaluation for pulmonary aspergillosis or mucormycosis at the two participating centers. By NGS platform, 483 cases (81.2%) underwent mNGS, 110 (18.5%) underwent tNGS, and platform information was missing for 2 cases. Bronchoalveolar lavage fluid (BALF) was the predominant sample type (Table 1A). BALF-GM was measured in 434 of the 595 NGS-tested cases (72.9%), defining the strict three-way subset used as the primary analytic population (NGS, BALF-GM and culture all available). Within this strict subset, NGS platform distribution was mNGS 404 (93.1%), tNGS 29 (6.7%), and missing 1 (0.2%); the marked predominance of mNGS in the strict subset reflects the local diagnostic ordering pattern. Severe pneumonia was present in 44.9% (267/595), immunocompromise in 34.5% (205/595), and in-hospital mortality was 14.0% (83/595).

**TABLE 1:** Cohort, same-subset positivity, and pairwise method overlap.

Item	Unit	Value
A. Cohort		
Total NGS-tested PAM cases	n	595
Sample type: BALF	n (%)	469 (78.8%)
Sample type: sputum	n (%)	126 (21.2%)
B. Same-subset positivity (n = 434)		
NGS Aspergillus-specific positivity	n (%)	353 (81.3%)
BALF-GM positivity (≥ 0.8)	n (%)	252 (58.1%)
Fungal culture positivity	n (%)	187 (43.1%)
C. Pairwise method overlap (n = 434)		
NGS^+^ / BALF-GM^+^	n	203
NGS^+^ / BALF-GM^–^	n	150
NGS^–^ / BALF-GM^+^	n	49
NGS^–^ / BALF-GM^–^	n	32
NGS^+^ / culture^+^	n	154
NGS^+^ / culture^–^	n	199
NGS^–^ / culture^+^	n	33
BALF-GM^+^ / culture^+^	n	139
BALF-GM^+^ / culture^–^	n	113
BALF-GM^–^ / culture^+^	n	48

### Method concordance (strict *n* = 434)

Within the strict 434-case subset, NGS Aspergillus positivity was 81.3% (353/434), BALF-GM (≥0.8) 58.1%, and culture 43.1% (Table 1B). Pairwise overlap was substantial but incomplete (Table 1C): NGS^+^/BALF-GM^–^ *n* = 150, NGS^–^/BALF-GM^+^ *n* = 49; NGS^+^/culture^–^ *n* = 199, NGS^–^/culture^+^ *n* = 33.

#### Clinical outcomes in the NGS-positive/culture-negative subset

Clinical outcome stratification across the four NGS x culture quadrants (strict subset, *n* = 434) revealed a graded mortality and severity pattern: NGS^+^/culture^+^ *n* = 154, mortality 25 (16.2%), severe 75 (48.7%); NGS^–^/culture^+^ *n* = 33, 8 (24.2%)/24 (72.7%); NGS^–^/culture^–^ *n* = 48, 9 (18.8%)/31 (64.6%); NGS^+^/culture^–^ *n* = 199, 25 (12.6%)/75 (37.7%). Within the 199 NGS^+^/culture^–^ cases, further GM stratification disclosed a marked outcome gradient consistent with a colonization-versus-disease interpretation: GM^+^/NGS^+^/culture^–^ (*n* = 83) mortality 19.3% and severe pneumonia 48.2%, versus GM^–^/NGS^+^/culture^–^ (*n* = 116) mortality 7.8% and severe pneumonia 30.2%; Fisher *p* = 0.018 (mortality)/*p* = 0.012 (severe pneumonia) between the two subgroups; Supplementary Table S5). This pattern is consistent with the GM-negative subset of NGS-positive/culture-negative cases containing a higher proportion of Aspergillus colonization rather than progressive disease. In addition, 196/199 (98.5%) of NGS-positive/culture-negative patients received antifungal therapy in routine practice (168 monotherapy, predominantly voriconazole; 28 combination), indicating that clinicians acted on NGS positivity as a treatment-triggering signal. Within this subset, GM-negative patients on antifungal monotherapy (*n* = 105) had in-hospital mortality of 4.8%. We caution explicitly against attributing this lower mortality solely to NGS-triggered antifungal therapy, as the GM-negative monotherapy subgroup is by definition enriched for lower fungal burden and milder baseline disease, which independently predict better outcomes; case-mix severity is therefore a major confounder, and disentangling its contribution from early NGS detection requires prospective follow-up. A clinically informative observation arose in the COPD subgroup (*n* = 31 NGS^+^/culture^–^, of which 23 GM^–^/NGS^+^/culture^–^): 100% (31/31) received triazole therapy with in-hospital mortality of 3.2% (1/31), markedly lower than the broader COPD cohort (13.3%), and attending physicians observed clinical resolution of bronchospasm following triazole initiation in cases otherwise refractory to bronchodilators—an unsystematic bedside response that aligns with the mortality pattern and supports the interpretation that NGS-detected Aspergillus in this host group reflects fungal airway involvement (chronic pulmonary aspergillosis, fungal tracheobronchitis, or low-burden invasive disease) rather than benign colonization.

Within these 199 NGS-positive/culture-negative cases, antifungal therapy was initiated in 196/199 (98.5%) patients in routine practice—168 (84.4%) received antifungal monotherapy (predominantly voriconazole, also isavuconazole or posaconazole), 28 (14.1%) received antifungal combination therapy, 1 was untreated and 2 had missing therapy records—indicating that clinicians in routine practice acted on NGS-positive/culture-negative Aspergillus signals as a treatment-triggering finding rather than as colonization. Cross-classified by BALF-GM and antifungal regimen, in-hospital mortality showed a striking gradient: GM^+^/monotherapy (*n* = 63) 17.5%, GM^+^/combination (*n* = 19) 26.3%, GM^–^/monotherapy (*n* = 105) 4.8%, GM^–^/combination (*n* = 9) 33.3% (Fisher exact, GM^–^/monotherapy vs. all other antifungal-treated strata, *p* = 0.0008; Supplementary Table S3). The GM^–^/NGS^+^/culture^–^ monotherapy subgroup—the largest single sub-stratum within the 199-case subset—thus exhibited the lowest mortality of any sub-stratum despite uniform antifungal treatment, a pattern more consistent with early or lower-burden true Aspergillus infection responsive to antifungal monotherapy than with a colonization-dominated population.

### GM-negative/NGS-positive phenotype

**FIGURE 3:**
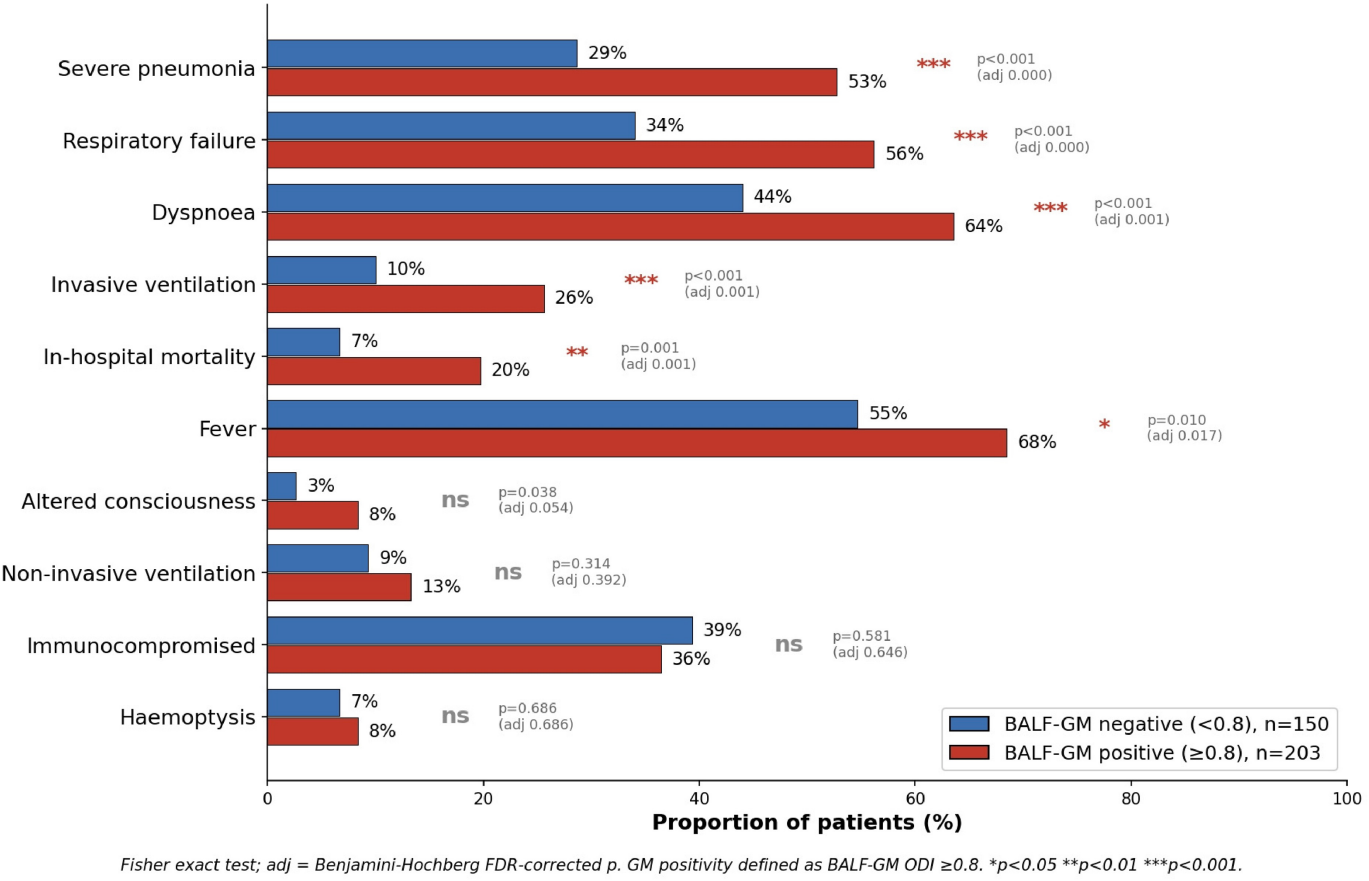
Clinical phenotype by BALF-GM status among NGS-Aspergillus-positive patients. Significance symbols defined (*p < 0.05, **p < 0.01, ***p < 0.001), based on Fisher exact tests with Benjamini-Hochberg FDR-corrected p values. Group labels and n updated to the final dataset (BALF-GM negative *n* = 150 versus BALF-GM positive *n* = 203; total *N* = 353); bars show GM-negative versus GM-positive NGS-positive groups.

GM-negative/NGS-positive cases (*n* = 150) showed a milder phenotype than GM-positive/NGS-positive cases (*n* = 203): severe pneumonia 29% vs. 53% (*p* = 1.6 × 10^−4^), mortality 7% vs. 20% (*p* = 8.4 × 10^−4^), with lower CRP, LDH, neutrophils, and higher albumin and lymphocytes (Table 2 and Figure 3).

**TABLE 2:** Clinical and laboratory features by NGS-Aspergillus and BALF-GM status (strict *n* = 434).

Variable	Type	GM^+^/NGS^+^	GM^–^/NGS^+^	GM^+^/NGS^–^	GM^–^/NGS^–^	p (4-grp)	p (GM^–^/NGS^+^ vs GM^+^/NGS^+^)
Age >65	binary	78/203 (38.4%)	55/150 (36.7%)	22/49 (44.9%)	15/32 (46.9%)	0.590	0.824
Immunocompromised	binary	74/203 (36.5%)	59/150 (39.3%)	21/49 (42.9%)	9/32 (28.1%)	0.550	0.581
Inhaled corticosteroids	binary	14/193 (7.3%)	19/149 (12.8%)	3/44 (6.8%)	8/32 (25.0%)	0.013	0.098
Leukemia	binary	7/203 (3.4%)	1/150 (0.7%)	0/49 (0.0%)	0/32 (0.0%)	0.135	0.145
Solid malignancy	binary	18/203 (8.9%)	17/150 (11.3%)	6/49 (12.2%)	1/32 (3.1%)	0.464	0.475
Chemotherapy	binary	17/203 (8.4%)	13/150 (8.7%)	6/49 (12.2%)	0/32 (0.0%)	0.269	1.000
Organ transplant	binary	10/203 (4.9%)	4/150 (2.7%)	5/49 (10.2%)	1/32 (3.1%)	0.173	0.410
Autoimmune disease	binary	29/203 (14.3%)	26/150 (17.3%)	7/49 (14.3%)	5/32 (15.6%)	0.880	0.460
COPD	binary	25/203 (12.3%)	29/150 (19.3%)	13/49 (26.5%)	8/32 (25.0%)	0.042	0.075
Asthma	binary	10/203 (4.9%)	12/150 (8.0%)	7/49 (14.3%)	3/32 (9.4%)	0.140	0.270
Interstitial lung disease	binary	31/203 (15.3%)	26/150 (17.3%)	5/49 (10.2%)	4/32 (12.5%)	0.646	0.661
Diabetes	binary	70/203 (34.5%)	36/150 (24.0%)	15/49 (30.6%)	14/32 (43.8%)	0.072	0.035
Severe pneumonia	binary	107/203 (52.7%)	43/150 (28.7%)	40/49 (81.6%)	15/32 (46.9%)	0.000	0.000
Respiratory failure	binary	114/203 (56.2%)	51/150 (34.0%)	40/49 (81.6%)	15/32 (46.9%)	0.000	0.000
Invasive ventilation	binary	52/203 (25.6%)	15/150 (10.0%)	24/49 (49.0%)	4/32 (12.5%)	0.000	0.000
Cavitation	binary	58/203 (28.6%)	33/150 (22.0%)	6/49 (12.2%)	9/32 (28.1%)	0.088	0.177
Consolidation	binary	106/203 (52.2%)	69/150 (46.0%)	28/49 (57.1%)	20/32 (62.5%)	0.258	0.282
Ground-glass opacity	binary	92/203 (45.3%)	62/150 (41.3%)	21/49 (42.9%)	10/32 (31.2%)	0.494	0.515
Pleural effusion	binary	58/203 (28.6%)	25/150 (16.7%)	21/49 (42.9%)	8/32 (25.0%)	0.002	0.011
Halo / crescent sign	binary	9/203 (4.4%)	3/150 (2.0%)	1/49 (2.0%)	1/32 (3.1%)	0.595	0.249
Tree-in-bud	binary	16/203 (7.9%)	16/150 (10.7%)	7/49 (14.3%)	4/32 (12.5%)	0.508	0.454
Bronchiectasis	binary	35/203 (17.2%)	24/150 (16.0%)	6/49 (12.2%)	5/32 (15.6%)	0.864	0.775
IPA subtype	binary	181/203 (89.2%)	125/150 (83.3%)	45/49 (91.8%)	26/32 (81.2%)	0.210	0.116
CPA subtype	binary	19/203 (9.4%)	16/150 (10.7%)	3/49 (6.1%)	6/32 (18.8%)	0.301	0.721
Mucormycosis (Rhizopus)	binary	25/203 (12.3%)	5/150 (3.3%)	0/49 (0.0%)	0/32 (0.0%)	0.000	0.003
Fungal bronchitis	binary	3/203 (1.5%)	8/150 (5.3%)	1/49 (2.0%)	0/32 (0.0%)	0.115	0.060
Culture positive	binary	120/203 (59.1%)	34/150 (22.7%)	19/49 (38.8%)	14/32 (43.8%)	0.000	0.000
Pathology positive	binary	3/203 (1.5%)	1/150 (0.7%)	0/49 (0.0%)	0/32 (0.0%)	0.671	0.640
IgG/IgE positive	binary	58/203 (28.6%)	26/150 (17.3%)	8/49 (16.3%)	15/32 (46.9%)	0.001	0.016
Bacterial co-detection (any test)	binary	131/203 (64.5%)	87/150 (58.0%)	39/49 (79.6%)	24/32 (75.0%)	0.027	0.224
Viral co-detection (any test)	binary	103/203 (50.7%)	53/150 (35.3%)	27/49 (55.1%)	19/32 (59.4%)	0.006	0.005
BALF sample (vs sputum)	binary	201/203 (99.0%)	150/150 (100.0%)	49/49 (100.0%)	32/32 (100.0%)	0.515	0.510
In-hospital mortality	binary	40/203 (19.7%)	10/150 (6.7%)	16/49 (32.7%)	1/32 (3.1%)	0.000	0.001
Age (years)	continuous	62.0 (53.0-69.0)	61.0 (50.2-69.0)	64.0 (59.0-71.0)	63.0 (55.8-70.2)	0.110	0.552
Length of stay (days)	continuous	11.0 (8.0-17.0)	11.0 (8.0-15.0)	15.0 (10.0-25.0)	11.0 (9.0-14.0)	0.006	0.415
PSI score	continuous	76.0 (57.5-102.0)	66.5 (52.0-80.0)	89.0 (70.0-113.0)	74.0 (50.5-86.5)	0.000	0.001
CURB score	continuous	1.0 (0.0-2.0)	1.0 (0.0-1.0)	2.0 (1.0-2.0)	0.5 (0.0-2.0)	0.000	0.000
WBC (10^∧^9/L)	continuous	8.7 (5.8-12.7)	7.2 (5.1-10.1)	8.5 (6.4-12.8)	8.3 (6.2-11.6)	0.002	0.000
Neutrophils	continuous	6.7 (4.0-11.6)	4.9 (3.1-7.7)	7.3 (4.4-11.1)	6.5 (3.8-9.1)	0.000	0.000
Lymphocytes	continuous	1.0 [0.7-1.4]	1.4 [0.8-1.8]	0.8 [0.5-1.3]	1.1 [0.7-1.6]	0.000	0.000
CRP	continuous	48.0 [11.5-119.2]	10.2 [2.5-64.6]	80.0 [20.4-144.2]	70.1 [19.0-160.0]	0.000	0.000
PCT	continuous	0.2 [0.2-0.3]	0.2 [0.2-0.2]	0.2 [0.2-0.4]	0.2 [0.2-0.3]	0.034	0.106
LDH	continuous	249.0 [180.5-405.5]	196.0 [160.0-271.2]	268.5 [195.2-426.0]	236.0 [183.5-284.5]	0.001	0.001
Albumin	continuous	35.2 [30.6-39.5]	37.8 [34.2-42.5]	35.3 [30.5-40.0]	38.2 [32.7-41.8]	0.000	0.000

### Multivariable predictors of GM-negative status

**FIGURE 4:**
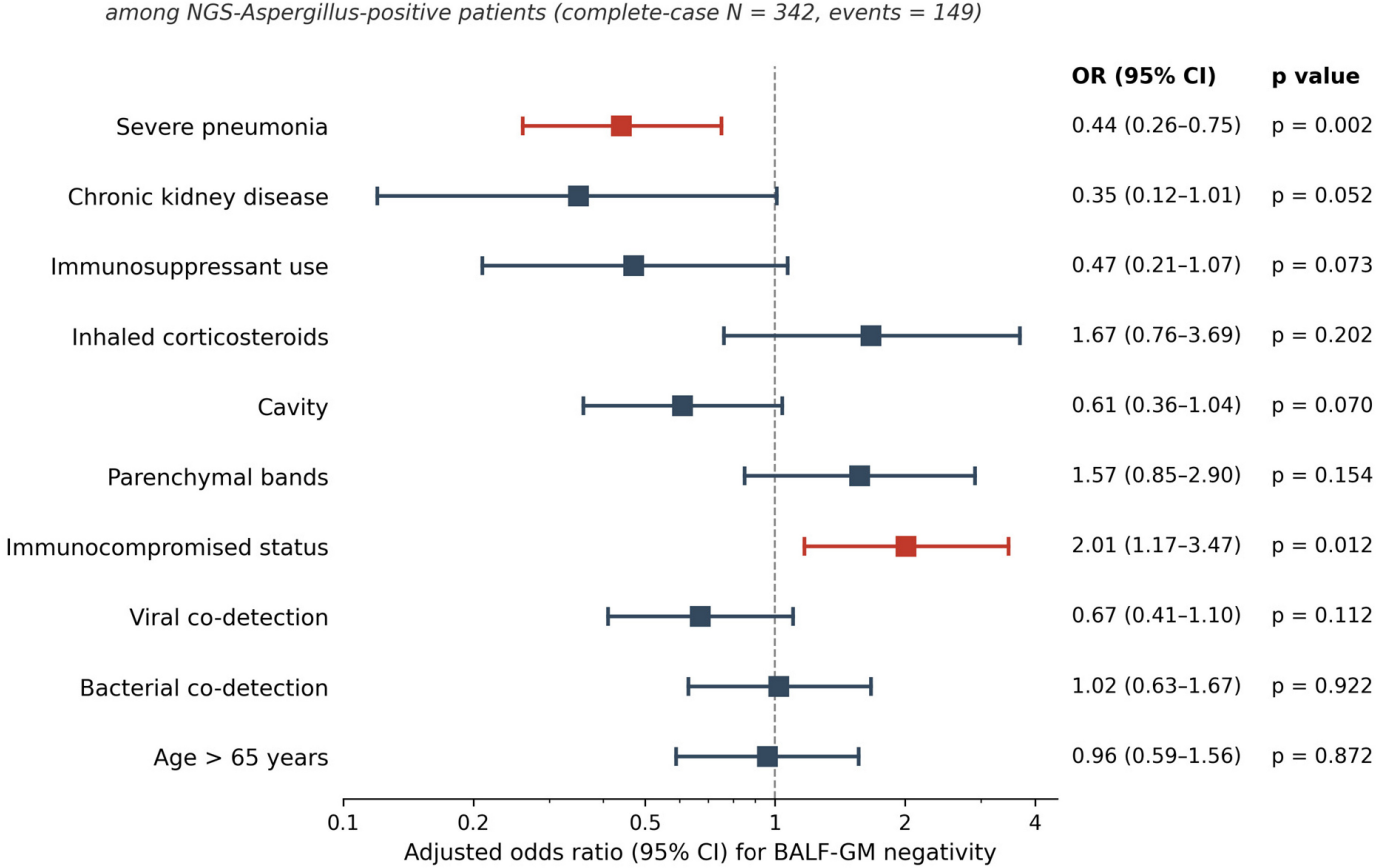
Forest plot now shows the final multivariable model: complete-case *N* = 342, events = 150, 10 covariates. Severe pneumonia adjusted OR 0.44 (0.26–0.75), *p* = 0.002; immunocompromised adjusted OR 2.01 (1.17–3.47), *p* = 0.012; chronic kidney disease adjusted OR 0.35 (0.12–1.01), *p* = 0.052 (non-significant trend, no longer significant after dataset correction); remaining covariates non-significant.

Among NGS-positive cases (univariate *n* = 353; complete-case *n* = 342), severe pneumonia was independently inversely associated with GM-negative status adjusted OR 0.44 (0.26–0.75, *p* = 0.002), and immunocompromise was independently positively associated (adjusted OR 2.01, 95% CI 1.17–3.47, *p* = 0.012); chronic kidney disease adjusted OR 0.35 (0.12–1.01, *p* = 0.052) (Table 3 and Figure 4). Mucormycosis was excluded from the multivariable model owing to near-perfect separation (univariate OR 0.05, *p* = 4.1 × 10^−3^).

**TABLE 3:** Univariate and multivariable logistic regression for GM-negative status among NGS-Aspergillus-positive PAM cases.

Predictor	Univariate OR	95% CI	p	Adjusted OR	95% CI	*p*
Age >65 years	0.93	0.60-1.44	0.736	0.96	0.59-1.56	0.872
Bacterial co-detection	0.76	0.49-1.17	0.212	1.02	0.63-1.67	0.922
Cavity	0.71	0.43-1.15	0.164	0.61	0.36-1.04	0.070
Chronic kidney disease	0.26	0.10-0.69	0.007	0.35	0.12-1.01	0.052
Immunocompromised	1.13	0.73-1.75	0.581	2.01	1.17-3.47	0.012
Immunosuppressant use	0.55	0.28-1.07	0.079	0.47	0.21-1.07	0.073
Inhaled corticosteroids	1.87	0.90-3.86	0.092	1.67	0.76-3.69	0.202
Parenchymal bands	2.09	1.18-3.68	0.011	1.57	0.85-2.90	0.154
Severe pneumonia	0.36	0.23-0.56	<0.001	0.44	0.26-0.75	0.002
Viral co-detection	0.53	0.34-0.82	0.004	0.67	0.41-1.10	0.112

Odds ratios >1 indicate higher odds of BALF-GM negativity; odds ratios <1 indicate higher likelihood of BALF-GM positivity. The multivariable model used *n* = 353 (univariate); complete-case *n* = 342, events = 150 and included 10 prespecified variables. Viral and bacterial co-detection were defined using total-pathogen variables, independent of NGS-only detection. Leukemia was not included in the main model because of the small number of cases, but was evaluated in Supplementary Table S4.

### NGS Aspergillus signal versus BALF-GM intensity

Within the mNGS-only subset of the strict cohort (*n* = 396; tNGS *n* = 29 was insufficient for stratified quantitative analysis), NGS Aspergillus positivity was essentially flat across BALF-GM intensity bands (80.2% at GM < 0.5; 85.0% at 0.5–0.99; 81.6% at 1.0–1.99; 77.8% at ≥ 2.0; χ^2^ *p* = 0.75; Supplementary Table S4). Aspergillus read counts (mNGS only, where reads were enumerated) showed a modest non-monotonic association across bands (Kruskal-Wallis *p* = 0.054) and a weak quantitative correlation with BALF-GM titer (Spearman ρ = 0.185, *n* = 246, *p* = 0.004; Supplementary Tables S1, S2). The weakness and directional ambiguity of this association—together with the small tNGS reads-available subset (*n* = 3) precluding pooled analysis—indicate that BALF-GM intensity and mNGS Aspergillus read magnitude capture only partly overlapping facets of fungal burden and should not be used as interchangeable quantitative indicators. Read-count data from mNGS and tNGS are not directly comparable due to fundamental differences in genomic coverage and library normalization; cross-technology pooling was not performed. The mNGS-stratified results are shown in Figure 1.

### GM × NGS quadrant analysis

**FIGURE 5:**
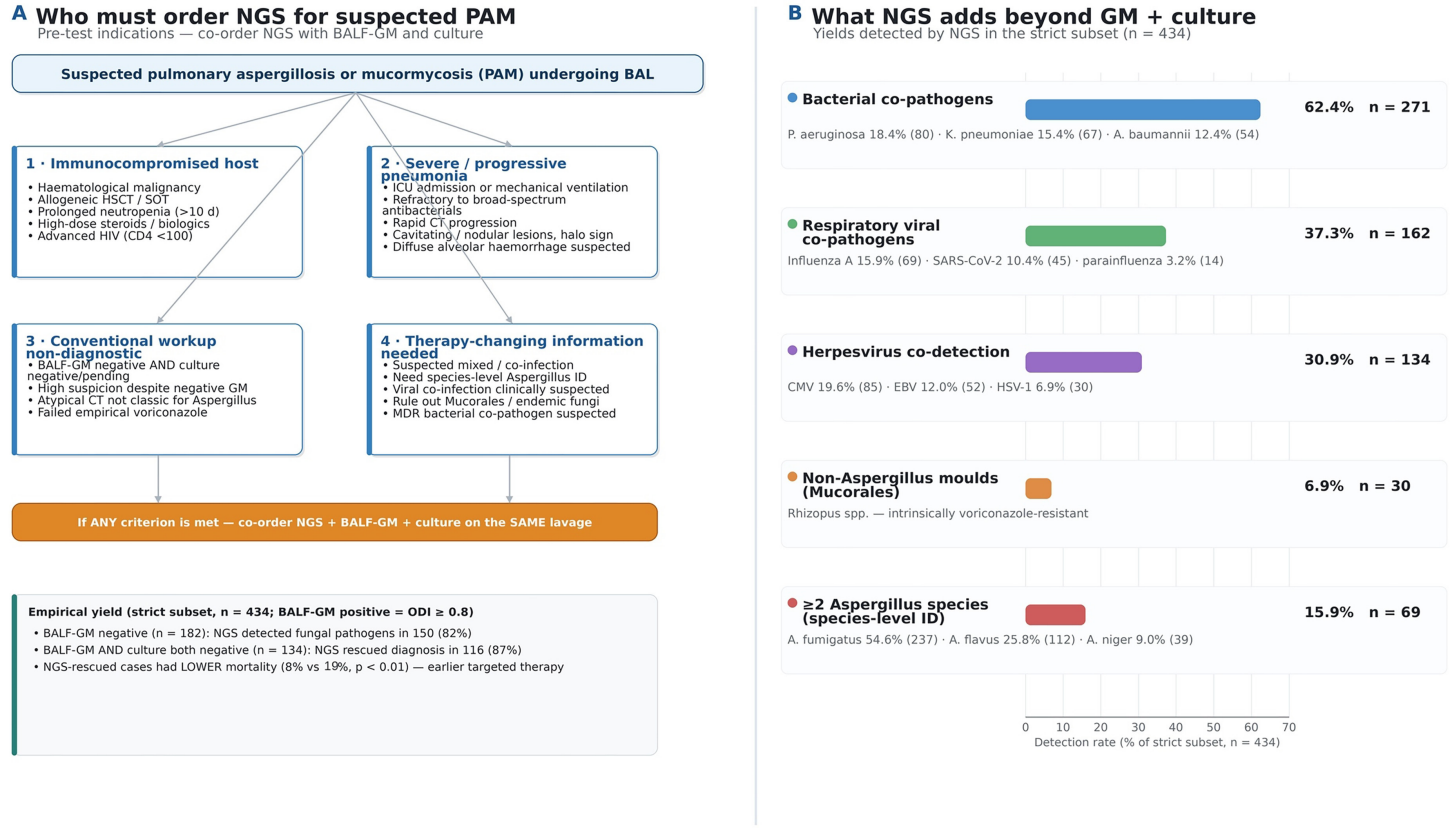
Who needs NGS for suspected PAM **(A)**, and what NGS adds beyond GM + culture **(B)** (NGS added value beyond GM + culture, strict 434-case subset) updated: bacterial 62.4% (271), respiratory viral 37.3% (162), herpesvirus 30.9% (134) now shown separately, > = 2 Aspergillus species 15.9% (69; NGS or culture), Mucorales 6.9% (30; intrinsically resistant to voriconazole).

Within the strict 434-case subset, BALF-GM (≥ 0.8) was positive in 252 cases (58.1%) and negative in 182 (41.9%). The four GM × NGS quadrants were: GM^+^/NGS^+^ *n* = 203 (46.8%), GM^+^/NGS^–^ *n* = 49 (11.3%), GM^–^/NGS^+^ *n* = 150 (34.6%), and GM^–^/NGS^–^ *n* = 32 (7.4%). Severity and mortality differed substantially across quadrants: severe pneumonia ranged from 29% (GM^–^/NGS^+^) to 82% (GM^+^/NGS^–^), and in-hospital mortality from 3% (GM^–^/NGS^–^) to 33% (GM^+^/NGS^–^) (all *p* < 0.001 across quadrants). Beyond pathogen detection, NGS uniquely captured ≥ 2 co-existing Aspergillus species in 69 cases (15.9%); A. fumigatus 54.6%, A. flavus 25.8%, A. niger 9.0%, Rhizopus in 30 cases (6.9%), respiratory viral 162 (37.3%), herpesvirus 134 (30.9%; CMV 19.6%, EBV 12.0%, HSV-1 6.9%), bacterial 271 (62.4%; Pa 18.4%, Kp 15.4%, Ab 12.4%), ≥2 Aspergillus 69 (15.9%), Rhizopus 30 (6.9%). Figure 5 summarizes these findings as a clinical decision tree applicable once the three results return together.

## Discussion

In this two-center PAM case cohort, mNGS/tNGS revealed a broad polymicrobial spectrum that extended well beyond fungal detection alone, and showed only partial overlap with conventional microbiology in the strict same-subset analysis (*n* = 434). Three findings have practical implications.

First, NGS is complementary, not replacement. Within the same NGS-tested population, NGS fungal-signal positivity exceeded culture and BALF-GM individually but only modestly exceeded the composite of non-pathology conventional microbiology. Substantial numbers of NGS^+^/conventional- and NGS^–^/conventional+ cases (Table 1C) demonstrate that these modalities are overlapping but not interchangeable. This supports complementarity rather than substitution, in line with recent meta-analyses (Chen et al., 2022; Guo et al., 2024), a multicenter randomized trial showing that mNGS-guided management improved outcomes in severe community-acquired pneumonia (Wu et al., 2025), and the ESCMID/ECMM/ERS principle that no single modality is sufficient for PAM diagnosis (Ullmann et al., 2018).

Second, the incremental value of NGS concentrates in immunocompromised, non-severe patients. The GM-negative/NGS-positive subgroup represents a large fraction of NGS-positive PAM and exhibits a consistently milder phenotype with lower mortality. After multivariable adjustment, severe pneumonia was inversely associated, and immunocompromise positively associated, with GM-negative status; the observed GM-negative rate stratified by immunocompromise × severe-pneumonia status (Supplementary Table S5) and the corresponding two-item bedside score (Supplementary Table S6) summarize this two-axis stratification. The clinical interpretation is intuitive: BALF-GM declares itself in patients with high antigen load and overt severity, whereas in immunocompromised hosts whose PAM presentation is more indolent, lower-burden, and less inflammatory, BALF-GM is more often falsely negative; NGS, detecting nucleic acid at lower thresholds, fills this gap. This clinical phenotype—immunocompromised but not severely ill—coincides with the scenario in which both BALF-GM and culture are most likely to be falsely negative, and therefore is the scenario in which NGS adds the most diagnostic value (Figure 5). Notably, 98.5% (196/199) of NGS-positive/culture-negative patients received antifungal therapy in routine practice, indicating that clinicians acted on NGS positivity as a treatment-triggering signal. Within this subset, GM-negative patients receiving antifungal monotherapy (*n* = 104) had the lowest in-hospital mortality (4.8%), in contrast to higher mortality in the GM-positive monotherapy stratum (17.5%, *n* = 63) and the combination-therapy strata (GM^+^ 26.3%, *n* = 19; GM^–^ 33.3%, *n* = 9; Fisher exact *p* = 0.0008 for GM^–^/monotherapy versus all other antifungal-treated strata). While these data are not a formal treatment-response analysis—they lack systematic capture of radiographic and microbiologic endpoints—the treatment-uptake rate and the mortality pattern together are more consistent with NGS-positive/culture-negative cases representing early or lower-burden true Aspergillus infection responsive to antifungals, than with a colonization-dominated population.

Third, NGS captures a partly independent dimension of fungal disease. Within the mNGS-only subset, NGS Aspergillus positivity was essentially flat across BALF-GM bands (79–82%), and the within-platform read–GM correlation was weak (Spearman ρ = 0.185, *n* = 246, *p* = 0.0036, Kruskal-Wallis *p* = 0.0082). We interpret this weak association cautiously: the read–GM relationship is exploratory and should not be over-read as a quantitative biomarker pairing. Rather, these findings indicate that BALF-GM intensity and NGS Aspergillus read magnitude reflect partly overlapping but non-interchangeable facets of fungal disease, supporting interpretive rather than ratio-based use of the two assays in routine practice.

Taken together, these findings reframe the clinical question for NGS in PAM. Because GM, NGS and culture are co-ordered on the same specimen in routine Chinese tertiary practice, the question is not whether NGS outperforms BALF-GM in head-to-head accuracy comparisons, nor when to add NGS—both assays are already on the bench. The actionable question is how to act on the combination of results once they return. The decision tree in Figure 5, derived from the strict 434-case same-specimen subset, translates the four GM × NGS quadrants into specific management actions and identifies the GM^–^/NGS^+^ quadrant—large (34.6%), immunocompromise-enriched, milder phenotype—as the setting in which NGS contributes the most incremental diagnostic yield.

This study has several limitations that frame how the results should be used. First, the study lacks a non-PAM control group; we therefore do not—and cannot—report classical diagnostic-accuracy metrics (sensitivity, specificity, predictive values) for NGS against a non-disease reference. Accordingly, the present work is positioned as an interpretive framework for combining BALF-GM, NGS and culture when all three are co-ordered on the same lavage specimen in routine Chinese tertiary PAM practice, not as a diagnostic-accuracy study. Second, the design is retrospective; we partly mitigated potential bias by restricting primary analyses to the strict 434-case subset with NGS, BALF-GM and culture all available. Third, NGS was performed across two manufacturers (KingMed Diagnostics for tNGS; Dinfectome Inc. for mNGS, plus < 10 mNGS cases from Weiyuan and Weiyan Biotechnology); within the strict subset, however, mNGS predominated (93.1%) and the vast majority of mNGS cases were supplied by Dinfectome. For analyses dependent on intra-platform read-count comparability we further restricted to Dinfectome cases, reducing vendor-source heterogeneity for the main quantitative analyses. Quantitative comparisons were stratified by platform and were never pooled across mNGS and tNGS, given fundamental differences in genomic coverage and library normalization. Fourth, within the strict subset, species-level Aspergillus read counts were available for 246 of 348 NGS-positive cases (70.7%; mNGS 76.9%, tNGS 10.3%); reads-missing cases were concentrated in earlier-period mNGS reports in which species-level reads were not enumerated in the free-text output, irrespective of parsing approach. To address parser fidelity, all qualitative organism flags from the full 595-case NGS report set were independently verified by two-author manual review, with 100% concordance with the automated parser. Fifth, structured follow-up data on antifungal-versus-antibacterial treatment response (radiographic resolution, microbiologic clearance, time-to-defervescence) were not uniformly captured in this retrospective two-center cohort, precluding a formal treatment-response analysis. To partially address this, we leveraged the available treatment-regimen and in-hospital outcome data: within the 199 NGS-positive/culture-negative cases, 98.5% received antifungal therapy in routine practice, and the GM-negative/monotherapy stratum showed the lowest in-hospital mortality (4.8%, *n* = 104) of any sub-stratum (Fisher exact *p* = 0.0008 versus all other antifungal-treated strata; see Results and Supplementary Table S3). This combined treatment-uptake and outcome evidence supports—but does not formally prove—that the GM-negative/NGS-positive/culture-negative subgroup is dominated by early or lower-burden true infection rather than colonization. A prospective sub-study capturing antifungal-versus-antibacterial treatment-response endpoints is identified as an important direction for future work. Sixth, the cohort is drawn from two Chinese tertiary centers, and external generalizability to other diagnostic settings or non-Chinese populations remains to be confirmed.

Interpreting combined GM + NGS results in routine practice. Because BALF-GM, NGS and culture are routinely co-ordered on the same lavage specimen in Chinese tertiary practice, the clinically actionable question is interpretive—how to act when the three results return together—rather than selective. Within the strict 434-case subset, the four GM × NGS combinations stratify cases into clinically distinct quadrants with markedly different severity (29–82% severe pneumonia) and mortality (3–33%). The GM^+^/NGS^–^ quadrant, although small (12%), is the most severely ill subgroup and is precisely where a negative NGS must NOT be used to rule out PAM; empirical antifungal therapy should proceed and re-examination for non-Aspergillus molds should be considered if response is poor. Conversely, the GM^–^/NGS^+^ quadrant (37%) is enriched for immunocompromised hosts, has a milder phenotype and lower mortality, and is the principal scenario in which NGS adds incremental diagnostic yield over conventional microbiology—a setting where confirming species, integrating imaging, and screening for Mucorales (Rhizopus accounted for 7% of the strict subset) should drive therapy selection. The double-positive (44%) and double-negative (8%) quadrants represent classical concordant PAM and low-yield molecular testing, respectively, where management follows established pathways. In all four quadrants, NGS contributes information beyond pathogen attribution alone: species-level Aspergillus identification (≥ 2 species in 16%), bacterial (62%) and respiratory viral (37%) co-pathogen detection that frequently alters empirical antibacterial and antiviral therapy, including timely identification of CMV, influenza A and SARS-CoV-2.

## Conclusion

When BALF-GM, NGS and culture are co-ordered together on the same lavage—as is standard in Chinese tertiary PAM practice—the practical task is interpreting the combination of results, not deciding whether to add NGS. The four GM × NGS quadrants (Figure 5) define clinically distinct phenotypes and actions, and across all quadrants NGS contributes species-level, mold-spectrum, and co-pathogen information that materially shapes empirical antifungal, antibacterial and antiviral therapy. We propose the GM × NGS decision tree, anchored in the strict same-specimen subset (*n* = 434), as a concise interpretive framework for routine PAM workup.

## Take-home message

In Chinese tertiary practice, BALF-galactomannan (GM), NGS and culture are routinely co-ordered on a single lavage specimen, so the clinical question is interpretive—how to act on the combination of results—rather than selective. In a two-center cohort, with all method comparisons restricted to the strict 434-case subset where the three assays were performed on the same specimen, the four GM × NGS combinations delineate clinically distinct phenotypes and actions: GM^+^/NGS^+^ (44%, mortality 20%)—standard concordant PAM, treat with voriconazole/isavuconazole and use NGS for species and co-pathogen detail; GM^+^/NGS^–^ (11%, mortality 33%)—most severe quadrant, treat empirically and do NOT use a negative NGS to rule out PAM; GM^–^/NGS^+^ (37%, mortality 7%)—highest NGS yield, often immunocompromised hosts, screen for Mucorales (Rhizopus captured in 7% of strict subset); GM^–^/NGS^–^ (8%, mortality 3%)—low molecular yield, re-evaluate alternative diagnoses. Across all quadrants, NGS adds species-level Aspergillus identification (≥ 2 species in 16%), Mucorales (7%), respiratory viral (37%) and bacterial (62%) co-pathogen detection that materially alter empirical therapy.

## Data Availability

The raw data supporting the conclusions of this article will be made available by the authors, without undue reservation.
