# Supplementary material for: Interpreting combined BALF-galactomannan and next-generation sequencing in pulmonary aspergillosis and mucormycosis: a two-center clinical decision framework
Source: Front Microbiol. 2026 Jul 22;17:1873197. doi: 10.3389/fmicb.2026.1873197 (PMC13440257; doi:10.3389/fmicb.2026.1873197)
Supplement: Supplementary file 1 [file Supplementary_file_1.docx]

Supplementary tables:

Table S1. NGS Aspergillus read counts stratified by BALF-GM intensity band (reads-available n = 246).

Table S2. Quantitative association between NGS Aspergillus reads and BALF-GM titre (mNGS only, n = 246).

Table S3. NGS Aspergillus positivity stratified by BALF-GM intensity band (mNGS only, n = 404).

Table S4. Leukemia sensitivity analysis for BALF-GM negativity.

Table S5. Treatment and outcomes by GM × NGS × culture stratum (strict 434-case subset).

Table S6. GM-negative rate by immunocompromise × severe pneumonia (n = 353).

Table S7. Two-item bedside score and observed GM-negative rate.

Supplementary Table S1. NGS Aspergillus read counts stratified by BALF-GM intensity band (reads-available n=246).

| **BALF-GM band** | **N** | **Median reads** |
| --- | --- | --- |
| <0.8 | 103 | 47 |
| 0.8–1.99 | 36 | 330 |
| ≥2.0 | 107 | 327 |

*Footnote. Aspergillus reads = per-case sum of species-level Aspergillus reads from the NGS report. Mucorales reads excluded. Kruskal–Wallis p=0.0082. Read-count analysis was restricted to cases with enumerated Aspergillus read counts.*

Footnote. Spearman ρ=0.185 (n=246, p=0.0036); log–log Pearson r=0.160 (p=0.0120). These correlations are weak and explicitly described in the manuscript as exploratory rather than as a quantitative biomarker relationship.

Supplementary Table S2. Quantitative association between NGS Aspergillus reads and BALF-GM titre (mNGS only, n=246).

| **Comparison** | **Test** | **Statistic** | **p-value** | **n** |
| --- | --- | --- | --- | --- |
| Aspergillus reads vs BALF-GM ODI | Spearman ρ | ρ=0.185 | 0.0036 | 246 |
| log10(Asp reads) vs log10(GM) | Pearson r | 0.160 | 0.0120 | 246 |
| Aspergillus reads across 3 GM bands | Kruskal–Wallis | — | 0.0082 | 246 |

Footnote. Spearman ρ=0.185 (n=246, p=0.0036); log–log Pearson r=0.160 (p=0.0120). These correlations are weak and explicitly described in the manuscript as exploratory rather than as a quantitative biomarker relationship.

Supplementary Table S3. NGS Aspergillus positivity stratified by BALF-GM intensity band (mNGS only, n=404).

| **BALF-GM band** | **N** | **NGS-Aspergillus positive n** | **NGS-Aspergillus positivity** |
| --- | --- | --- | --- |
| <0.8 | 163 | 132 | 81.0% |
| 0.8–1.99 | 61 | 50 | 82.0% |
| ≥2.0 | 180 | 142 | 78.9% |

*Footnote. NGS-Aspergillus qualitative positivity was essentially flat across BALF-GM ODI bands (78.9–82.0%; chi-square p=0.828).*

Supplementary Table S4. Leukemia sensitivity analysis for BALF-GM negativity among NGS-Aspergillus-positive cases.

| **Variable** | **n** | **GM-negative n (%)** | **GM-positive n (%)** | **Univariate p** | **Adjusted OR** | **95% CI** | **Adjusted p** |
| --- | --- | --- | --- | --- | --- | --- | --- |
| Leukemia | 8 | 1/8 (12.5%) | 7/8 (87.5%) | 0.145 | 0.12 | 0.01-1.10 | 0.061 |

*Footnote. Leukemia was added to the Figure 4 main multivariable model as a sensitivity analysis. The direction suggests a non-significant trend toward BALF-GM positivity, but the estimate is unstable because leukemia cases were few (n=8).*

Supplementary Table S5. Treatment and outcomes by GM x NGS x culture stratum within the 199 NGS-positive/culture-negative subset (strict 434-case subset)

**Part A. In-hospital mortality and severe pneumonia by NGS x culture quadrant (strict 434-case subset)**

Supplementary Table S5, Part A. In-hospital mortality and severe pneumonia by NGS × culture quadrant (strict 434-case subset).

| **NGS x culture stratum** | **n** | **Mortality, n (%)** | **Severe pneumonia, n (%)** |
| --- | --- | --- | --- |
| NGS^+^/culture^−^ | 199 | 25 (12.6) | 75 (37.7) |
| NGS^+^/culture^+^ | 154 | 25 (16.2) | 75 (48.7) |
| NGS^−^/culture^+^ | 33 | 8 (24.2) | 24 (72.7) |
| NGS^−^/culture^−^ | 48 | 9 (18.8) | 31 (64.6) |

Part B. Treatment regimen and outcomes within the 199 NGS-positive/culture-negative subset, stratified by BALF-GM (cut-off 0.8) and antifungal regimen

Supplementary Table S5, Part B. Treatment regimen and outcomes within the 199 NGS-positive/culture-negative subset.

| **BALF-GM stratum** | **Antifungal regimen** | **n** | **Mortality, n (%)** | **Severe pneumonia, n (%)** |
| --- | --- | --- | --- | --- |
| GM^+^ | Antifungal monotherapy | 63 | 11 (17.5) | 27 (42.9) |
| GM^+^ | Antifungal combination | 19 | 5 (26.3) | 12 (63.2) |
| GM^+^ | Therapy data missing | 1 | 0 (0.0) | 1 (100.0) |
| GM^−^ | Antifungal monotherapy | 105 | 5 (4.8) | 29 (27.6) |
| GM^−^ | Antifungal combination | 9 | 3 (33.3) | 4 (44.4) |
| GM^−^ | No antifungal therapy | 1 | 0 (0.0) | 1 (100.0) |
| GM^−^ | Therapy data missing | 1 | 1 (100.0) | 1 (100.0) |
| All 199 | Antifungal therapy initiated | 196 | 24 (12.2) | 72 (36.7) |
| All 199 | No antifungal therapy or missing | 3 | 1 (33.3) | 3 (100.0) |

BALF-GM stratum defined by BALF-galactomannan ODI cut-off 0.8. Antifungal monotherapy predominantly voriconazole (n=146), with smaller numbers of isavuconazole, posaconazole, and itraconazole; antifungal combination most often voriconazole or isavuconazole plus amphotericin B. Strict 434-case analytic subset = adults with BALF specimens having co-ordered NGS and BALF-GM. Fisher exact test, GM−/monotherapy mortality versus all other antifungal-treated strata: p = 0.0008. NGS, next-generation sequencing; GM, galactomannan; BALF, bronchoalveolar lavage fluid; ODI, optical density index.

Supplementary Table S6. GM-negative rate by immunocompromise × severe pneumonia (n=353).

| **Immunocompromised** | **Severe pneumonia** | **N** | **GM-negative n** | **GM-negative rate** |
| --- | --- | --- | --- | --- |
| No | No | 140 | 73 | 52.1% |
| No | Yes | 80 | 18 | 22.5% |
| Yes | No | 63 | 34 | 54.0% |
| Yes | Yes | 70 | 25 | 35.7% |

*Footnote. Logistic-regression interaction term immunocompromise × severe pneumonia: p=0.47.*

Supplementary Table S7. Two-item bedside score (immunocompromise + non-severe) and observed GM-negative rate (n=353).

| **Score** | **N** | **GM-negative n** | **GM-negative rate** |
| --- | --- | --- | --- |
| 0 | 80 | 18 | 22.5% |
| 1 | 210 | 98 | 46.7% |
| 2 | 63 | 34 | 54.0% |

*Footnote. AUC for predicting GM-negative status = 0.60. Descriptive only.*
